# Antithyroid drugs and birth defects

**DOI:** 10.1186/s13044-020-00085-8

**Published:** 2020-06-27

**Authors:** Stine Linding Andersen, Stig Andersen

**Affiliations:** 1 Department of Clinical Biochemistry, Aalborg University Hospital, Hobrovej 18-22, 9000 Aalborg, Denmark; 2 Department of Clinical Medicine, Aalborg University, 9000 Aalborg, Denmark; 3 Department of Clinical Biochemistry, Viborg Regional Hospital, 8800 Viborg, Denmark; 4 Department of Geriatrics, Aalborg University Hospital, 9000 Aalborg, Denmark

**Keywords:** Pregnancy, Hyperthyroidism, Graves’ disease, Birth defects, Congenital malformations, Antithyroid drugs, Methimazole, Carbimazole, Propylthiouracil

## Abstract

Antithyroid drugs (ATDs) are preferred for the treatment of hyperthyroidism caused by Graves’ disease in pregnant women. The drugs have been a recognized treatment for decades, and a general risk of side effects is known. For the use of ATDs in pregnancy, a concern about teratogenic side effects has been brought forward since the 1970s. In more recent years, a number of large observational studies have added new evidence and quantified the risk of birth defects associated with different types of ATDs. The findings that both Methimazole (MMI) and Propylthiouracil (PTU) are associated with birth defects have challenged the clinical recommendations on the treatment of hyperthyroidism in pregnancy, and certain aspects remain unclarified. In this review, the current evidence on the risk of birth defects associated with the use of ATDs in early pregnancy is described, and determinants of causality are discussed. This includes the current evidence of a biological gradient and the role of maternal thyroid function per se*.* Finally, clinical aspects of the timing and type of treatment is discussed, and future perspectives are addressed. Current evidence corroborates a risk of birth defects associated with MMI while more evidence is needed to determine the teratogenic potential of PTU. Detailed assessment of type and timing of exposure in large cohorts are needed. Moreover, studies investigating alternative or new treatments are warranted.

## Introduction

Antithyroid drugs (ATDs) have been used in clinical practice for the treatment of hyperthyroidism for more than half a century and constitute a recognized treatment in non-pregnant and in pregnant individuals [1]. In pregnancy, the hyperthyroidism of Graves’ disease (GD) should be treated to prevent maternal and fetal complications, and ATDs are the treatment of choice [2–4]. Radioiodine should not be used in pregnancy, and surgery is preferably avoided [2–4]. A long known and general concern about the use of ATDs is the risk of side effects [5]. The most common side effects are less severe and typically cutaneous reactions [5]. On other hand, severe side effects related to treatment with ATDs such as agranulocytosis and liver failure are rare [5]. For the use of ATDs in pregnancy, adverse side effects may affect not only the pregnant woman, but also the developing fetus [6]. Increasing evidence has pointed towards a risk of birth defects associated with the use of ATDs for the treatment of hyperthyroidism in pregnant women, and the findings that a teratogenic risk may apply to all available ATDs challenge the clinical recommendations [2–4].

In this review, the current state of the art on the risk of birth defects associated with maternal use of ATDs in pregnancy is described, and methodological aspects are discussed. Furthermore, the review specifically addresses the existing knowledge on determinants of causality including the impact of the dose of ATDs and maternal thyroid function per se*.* Finally, perspectives on the timing of exposure and alternative or new treatments in pregnancy are introduced.

### ATDs and birth defects

ATDs are thionamides that block the synthesis of thyroid hormones [5]. The facts that the drugs are not naturally occurring in humans and that they interrupt an endogenous synthesis raise a concern about adverse effects. The available ATDs include Methimazole (MMI) and its prodrug Carbimazole (CMZ) as well as Propylthiouracil (PTU) [1]. In non-pregnant individuals, MMI is preferred for initial treatment due to the pharmacokinetic profile and less severe side effects [2, 4]. The initial choice of treatment differs in pregnant women, and PTU is recommended for the treatment of hyperthyroidism in early pregnancy due to a risk of birth defects associated with the use of MMI [2–4].

**Fig. 1:**
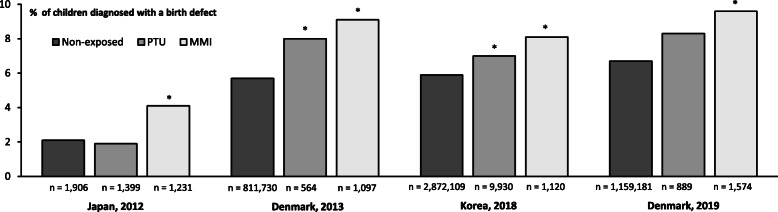
Main results from the observational studies [13–16] that detected an association between the use of antihyroid drugs in early pregnancy and birth defects. The figure illustrates the prevalence of birth defects in non-exposed children and in children exposed to maternal treatment with Propylthiouracil (PTU) or Methimazole (MMI) in early pregnancy. *indicates statistical significant difference for comparison to the non-exposed group

**Fig. 2:**
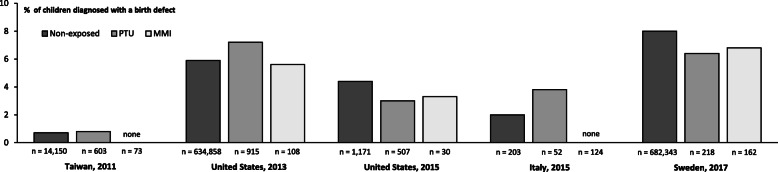
Main results from the observational studies [17–21] that found no association between the use of antihyroid drugs in early pregnancy and birth defects. The figure illustrates the prevalence of birth defects in non-exposed children and in children exposed to maternal treatment with Propylthiouracil (PTU) or Methimazole (MMI) in early pregnancy

A possible link between the use of ATDs and birth defects was first described 50 years ago. From the early report in 1972 [7], case reports and case series encircled a pattern of severe malformations after maternal use of MMI in early pregnancy including aplasia cutis, choanal atresia, esophageal atresia, omphalocele, and omphalomesenteric duct anomalies [8]. These findings led to the proposal of an MMI embryopathy in the 1990s [9]. Consequently, PTU was recommended for the treatment of maternal hyperthyroidism in the beginning of the twenty-first century, but uncertainties on the teratogenic profile of MMI prevailed [10, 11]. Another decade went past before the first observational studies that included non-exposed control groups were published [12]. During the 2010s, a number of observational studies have emerged that evaluated the association between exposure to ATDs in early pregnancy and birth defects (Figs. 1 and 2). The studies cover different populations in Asia (Taiwan [17], Japan [13], Korea [14]), Europe (Denmark [15, 16], Sweden [18], Italy [19]), and the United States (US) [20, 21]. Some studies found an association (Fig. 1), while others did not (Fig. 2). As previously discussed, a number of methodological differences between the studies may explain this disparity [12]. Firstly, the studies from Japan, Denmark, and Korea that detected an association included a large number of exposed children (Fig. 1). All these studies included more than 1000 MMI-exposed children and found that exposure to MMI was associated with a higher risk of birth defects (Fig. 1). An important similarity among the studies that observed an association with MMI (Fig. 1) was a risk of the severe malformations previously described as part of the MMI embryopathy. In contrast, the studies that did not detect an association with MMI included less than 200 exposed children (Fig. 2), but it is to be noted that more than half of the studies that found no association with either MMI or PTU included more the 500 PTU-exposed children. As discussed in further detail below, other determinants besides the number of exposed children may be of importance when evaluating and comparing study results. This includes the selection and definition of the non-exposed group, the method used for assessment of exposure and outcome, the timing of exposure and the age of the child at follow-up for the diagnosing of birth defects.

Considering PTU, this drug had not been linked to birth defects by the turn of the century [11] and the majority of later observational studies found no association between maternal use of PTU in early pregnancy and birth defects including the large study from Japan published in 2012 (Figs. 1 and 2). On the other hand, the subsequent studies from Denmark in 2013 and Korea in 2018 eported that PTU was associated with a higher frequency of birth defects as opposed to non-exposed (Fig. 1). The finding that PTU was associated with birth defects was new and challenged the clinical guidelines on which drug to use and further studies were warranted to corroborate the findings. A follow-up to the Danish study was published in 2019 that extended the cohort to recent years and included children born from 1997 to 2016. In this extended cohort, an association between the use of ATDs in early pregnancy and birth defects was substantiated (Fig. 1). Thus, MMI was associated with a higher risk of birth defects in a number of organ systems and with the severe malformations previously described. A higher frequency of birth defects after exposure to PTU was also observed (Fig. 1), but the association was at the border of statistical significance. Notably, an association with the subtypes of birth defects previously associated with PTU was still observed in the extended Danish cohort and supports an association [16].

When considering the findings and the disparity in results from different studies, details of outcome assessment may be of importance. Birth defects, also known as congenital malformations, are by definition present at birth [22]. However, this does not imply that all defects are diagnosed at birth. Firstly, none of the observational studies illustrated in Figs. 1 and 2 included data on prenatally diagnosed malformations from the use of ultrasound in pregnancy. The lack of such information may bias the associations observed particularly for the more severe malformations that could lead to the termination of a pregnancy. Secondly, some birth defects may not manifest until later in life and whether and when a malformation is diagnosed will depend on when the child is examined and what the examiner is looking for. Thus, it should be expected that particularly less severe malformations are diagnosed with a delay. In general, the prevalence of birth defects is 3% at birth increasing to 6% at two years of age [22]. Notably, the reported prevalence of birth defects among non-exposed children ranged from 0.7 to 8.0% in the observational studies investigating the association between maternal use of ATDs in early pregnancy and birth defects (Figs. 1 and 2). This may be partly due to the different follow-up time for diagnosis of birth defects (ranging up to the age of two years), but may also reflect other disparities (maternal report or examination by medical doctor, in- and/or outpatient diagnoses, types of malformations) [12]. The studies from Denmark [15, 16] and Korea [14] were register-based studies, and the children were followed to the age of two and one year of age, respectively, for any diagnoses of birth defects. In these studies [14–16], the type of malformations associated with exposure to PTU differed from those observed after MMI, and a detailed examination of these birth defects in the Danish study showed that they were confined to the face and neck region and the urinary system, and they appeared less severe [23]. On the other hand, the birth defects observed after exposure to PTU in the study from Korea [14] were confined to the urinary system and the musculoskeletal system, whereas no association with malformations of the face and neck region was observed. The study by Korelitz et al. from the US published in 2013 included 915 PTU-exposed children (Fig. 2). No overall association with PTU was observed in this study, but considering subtypes of birth defects results indicated a higher risk of malformation of the urinary system after exposure to PTU. The Danish [15, 16] and the Korean [14] studies both used the 10th revision of the International Classification of Disease (ICD) to identify birth defects in the child, whereas the US study [20] used the 9th revision of the ICD. As specified above, the age at follow-up differed between the studies. Furthermore, malformation of the face and neck region were classified together with those of the eye and the ear in the Korean study [14], whereas these malformation were evaluated in separate groups in the Danish studies [15, 16] and not specifically evaluated in the US study [20]. Adding to these considerations, the subtypes of malformations are rare, and it may be speculated if disparities in registration practice exist. Overall, the findings from different populations emphasize that further studies with more cases are needed to settle the teratogenic role of PTU, and the low prevalence of subtypes of birth defects call for large studies and extended follow-up.

### On the biological gradient

Strength, consistency and specificity are some of the key determinants of causality in observational studies [24]. Another determinant is the presence of a biological gradient in the associations observed [24]. As mentioned above, one of the methodological challenges when studying the association between in utero exposure and birth defects is the low prevalence of subtypes of malformations. This necessitates large study populations to obtain a sufficient number of exposed cases. Nationwide health registers available in different countries constitute a unique data source suitable for this purpose while providing a large study population and a low risk of selection bias. On the other hand, such registers are secondary research data meaning they are typically not collected with the primary intention of doing research [25]. This procedure may limit the available information of exposure and outcome. More detailed information is often available in studies that rely on the review of medical records, but this methodology often applies to a smaller study population, and the inclusion of patients from a specialized hospital department may inherit a risk of selection bias [26, 27]. Another methodological aspect is that the type of non-exposed control group differs between studies. Some studies included women with Graves’ disease who did not receive ATD in the pregnancy, while others attempted to include ‘thyroid healthy’ controls. This diversity in the choice of comparison group may influence study results and conclusions and challenge the assessment of the role ATD as opposed to other exposures related to maternal thyroid disease and thyroid autoimmunity.

Considering information on the dose of ATDs used for the treatment of hyperthyroidism in early pregnancy, this information was reported in a subset of observational studies (Table 1). The two studies form Japan [10, 13] relied on the review of medical records and found no evidence of a dose-dependent association. The study by Momotani et al. [10] (Table 1) included 643 children born to mothers with GD in the period from 1965 to 1980. A total of 243 children had been exposed to maternal use of MMI in the pregnancy and two of these children had a birth defect. Details on the maternal MMI treatment revealed that these children were born to mothers treated with a daily dose of 5 and 20 mg MMI, respectively, and a cumulative dose of 160 and 240 mg. The mean dose of maternal MMI treatment among the 241 exposed non-cases (children with no birth defect) was not reported, but it was concluded from these limited data that there was no dose-dependency of MMI on the occurrence of malformations [10]. In the more recent and larger study from Japan published in 2012 by Yoshihara et al. [13] (Table 1), it was reported that the mean dose of MMI among all exposed pregnancies was 5 mg/day (standard deviation: 8.1 mg/day) and that the dose of MMI did not differ between cases of birth defects (*n* = 50) and controls (*n* = 1181), *p* = 0.13. Notably, it appeared from the description of individual cases that the mean daily dose of MMI was 9 mg in cases of aplasia cutis (*n* = 8), 17 mg in omphalocele (n = 8), and 13 mg in cases of omphalomesenteric duct anomalies (*n* = 8), but no measure of comparison to exposed non-cases was reported [13]. The study from Korea [14] (Table 1) was a large, nationwide study and used data from the Korean National Health Insurance database, which included a measure of the cumulative dose of MMI and PTU during the first trimester of pregnancy. A dose-dependent relationship was reported for MMI in which case the prevalence of birth defects was higher with a cumulative dose of > 495 mg (*n* = 36 of 278; 13.0%) as compared to 1–126 mg (*n* = 21 of 282; 7.5%); adjusted odds ratio 1.87 (95% confidence interval (CI): 1.06–3.30). This was not the case for PTU with a similar prevalence of birth defects among children exposed to a cumulative dose of > 8600 mg (*n* = 159 of 2466; 6.5%) as compared to 1–1850 mg (*n* = 184 of 2502; 7.4%); adjusted odds ratio 0.90 (95% CI: 0.72–1.12) [14]. No information on the mean daily dose of MMI and PTU was reported, but when the cumulative doses reported are considered relatively to the duration of a first trimester, the dose-dependent relationship appears to be present even when relatively small doses of MMI are compared. This is the first study [14] to suggest a biological gradient in the associations observed, and it is a notable finding that dose-dependency was observed for MMI, but not for PTU. However, there are difficulties related to the assessment of a dose-dependent effect since the individual dose of ATD may vary by week of pregnancy and the indirect measure of exposure from redeemed prescriptions of drugs bring about some uncertainty on the exact timing and dose of exposure. Further studies are warranted to extend the findings including studies with detailed information on the daily dose, exact timing of exposure and a wider range of doses.

**Table 1:** Observational studies on the association between the dose of Methimazole used in early pregnancy and outcome of birth defects

Author	Year	Country	Data source	Exposed (n)^a^	Dose definition	Association^b^
Momotani et al. [10]	1984	Japan	Medical records	243	Cumulative dose (range: 10–1680 mg) Daily dose (categories: 5, 10, 15, 20, and ≥ 30 mg)	No
Yoshihara et al. [13]	2012	Japan	Medical records	1231	Daily dose (mean: 5 mg/day, standard deviation: 8.1 mg)	No
Seo et al. [14]	2018	Korea	Nationwide registers	1120	Cumulative dose (categories: 1–126, 127–260, 261–495, and > 495 mg)	Yes

^a^Number of children exposed to maternal use of Methimazole in the early pregnancy

^b^Indicates whether a dose-dependent association between Methimazole and birth defects was observed, see text for details

### On thyroid function per se

Another key aspect to consider in terms of causality from observational findings is analogy [24]. Namely, to address the likelihood of other possible explanations in the associations observed. For the association between ATDs and birth defects, the role of maternal thyroid function should be considered. As with details on treatment dose, information on maternal thyroid function in pregnancy is not always obtainable, especially when secondary data sources are used [25]. Data on maternal thyroid function in pregnancy were available and reported in a subset of the observational studies on the use of ATDs and birth defects (Table 2). The study by Momotani et al. [10] published in 1984 concluded that maternal hyperthyroidism per se was the main determinant of birth defects in the offspring, whereas a smaller study from Italy [19] and the larger studies from Japan [13] and Denmark [15] found no evidence of an association between maternal thyroid function and birth defects. Notably, the methods used for assessment of maternal thyroid function in pregnancy differed between the studies including the type of assay and the reference ranges used for classification for maternal thyroid function (Table 2). Furthermore, the subtype of maternal thyroid dysfunction, the timing of exposure in relation to the early pregnancy period and the choice of non-exposed control group differed and limits the possibility to draw definite conclusions.

**Table 2:** Observational studies on maternal thyroid function in early pregnancy and outcome of birth defects

Author	Year	Country	Sample	Exposed (n)^a^	Thyroid function tests	Assays	Reference range	Association^b^
Momotani et al. [10]	1984	Japan	Clinical	167	Free T4 index^c^	Not specified	Not specified	Yes
Yoshihara et al. [13]	2012	Japan	Clinical	Not specified	Free T4	Lumipulse, Fuji Rebio or ECLusys, Roche Diagnostics	Trimester	No
Gianetti et al. [19]	2015	Italy	Clinical	55	Free T4, free T3 and TSH	FT4 kit and FT3 kit, Technogenetics Delfia hTSH, Pharmacia	Not specified	No
Andersen et al. [16]	2019	Denmark	Biobank	951	Free T4 and TSH	Dimension Vista, Siemens Healthineers	Pregnancy week	No
Andersen et al. [16]	2019	Denmark	Biobank	2183	Free T4 and TSH	Advia Centaur XP, Siemens Healthineers	Pregnancy week	No

^a^Number of children exposed to abnormal maternal thyroid function in the early pregnancy

^b^Indicates whether an association between abnormal maternal thyroid function and birth defects was observed, see text for details

^c^In some cases the total T4, T3 and/or PBI concentration was measured

The study by Momotani et al. [10] (Table 2) included pregnant women with GD from a hospital clinic and in the majority of cases, a free T4 index was used for assessment of maternal thyroid function. No further details on the assay, the reference ranges used, and the timing of sampling in pregnancy were reported, but it was stated that the influence of pregnancy on the normal range of thyroid function tests was considered. In this study [10], the frequency of birth defects was higher in hyperthyroid women (*n* = 5 of 167; 3.0%) than in euthyroid women (*n* = 1 of 476; 0.2%), *p* < 0.01, however, the number of exposed cases was small, and the low prevalence of birth defects in non-exposed children is not in accordance with findings in later thorough studies. In the later and larger Japanese study by Yoshihara et al. [13] (Table 2), the assessment of maternal thyroid function in patients with GD was made from free T4 in the first trimester (after the first trimester for a subgroup of the patients) using two different automatic immunoassays, and results were evaluated according to a uniform trimester-specific reference range. In this study [13], the impact of maternal thyroid function was assessed in the non-exposed group, in the MMI-exposed group, and in the PTU-exposed group, respectively. No difference in the frequency of birth defects was observed when stratified by maternal hyperthyroidism within these groups, and in an adjusted model, maternal thyroid function did not associate with birth defects (adjusted odds ratio: 0.86 (95% CI: 0.63–1.1) [13]. In the study from Italy [19], 176 pregnant women with GD were treated with ATDs in pregnancy and had thyroid function assessed monthly. A total of 55 women were classified as hyperthyroid, because biochemical hyperthyroidism was seen twice during the pregnancy. For comparison, 121 euthyroid women treated with ATD were included as were 203 euthyroid pregnant women diagnosed with other subtypes of thyroid disease. No measure of comparison was provided, but it was concluded that the rate of malformations was not higher in any of the groups investigated than in the general population.

The most recent study from Denmark [16] differed from the Japanese studies [10, 13] and from the Italian study [19] in terms of the study population and the assessment of maternal thyroid function (Table 2). This study [16] included the measurement of TSH and free T4 in two independent cohorts using stored blood samples from 7624 pregnant women enrolled in the Danish National Birth Cohort (DNBC) and from 14,483 pregnant women enrolled in the North Denmark Region Pregnancy Cohort (NDRPC). Thus, these study populations were not confined to women with GD from a hospital department. Furthermore, pregnancy-week specific reference ranges established within each cohort were used for assessment of maternal thyroid function in the early pregnancy (median week 9–10). Overall, no association between maternal thyroid function and birth defects was observed in these cohorts (DNBC: adjusted hazard ratio 1.02 (95% CI: 0.86–1.22), NDRPC: 1.03 (0.82–1.30)). For subtypes of maternal thyroid dysfunction, no association between hyperthyroidism and birth defects was observed. However, in one of the cohorts, maternal overt hypothyroidism was a risk factor for birth defects in the child (adjusted hazard ratio: 1.91 (95% CI: 1.12–3.25), and this associated was dominated by malformations of the heart [16]. In the Japanese study from 2012 [13], it was also reported that the frequency of birth defects was higher in hypothyroid women compared to euthyroid women, and other observational studies investigating maternal thyroid function in pregnancy, but not exposure to ATDs, have indicated that maternal hypothyroidism may be a risk factor for birth defects including congenital heart defects [28–31]. However, studies are heterogeneous in the definition of exposure and outcome, and the underlying mechanisms are not clear. Thus, further studies are needed to address the association between maternal thyroid function in early pregnancy and birth defects including the differential exposure to ATDs and/or abnormal thyroid function and the potential risk of side effects related to over- and under-treatment of maternal thyroid disease.

### Timing and type of treatment

Results of the large observational studies published during the last decade have substantiated a concern about teratogenic side effects to the use of all ATDs in early pregnancy. Such findings challenge the clinical practice and have led to a re-evaluation of the clinical recommendations [2–4]. In the current guidelines [2–4], PTU is recommended for the treatment of hyperthyroidism in pregnancy with a focus on early pregnancy detection and early shift from MMI to PTU. Furthermore, the findings that all clinically available ATDs may be associated with birth defects have led to considerations on the timing and type of treatment [8]. This includes the benefits and risks of definitive treatment prior to pregnancy or a withdrawing of ATDs during the early pregnancy weeks in appropriately selected patients as well as considerations on alternative treatment options.

Considering the timing of exposure, a subset of the observational studies performed included a group of children considered as exposed to both MMI and PTU in early pregnancy (Table 3). Half of the studies reported that such ‘double exposure’ was associated with a higher risk of birth defects as compared to non-exposed (Table 3). The number of exposed children was limited in the majority of studies (Table 3), but the identification of these pregnancies is of clinical importance considering the timing of a shift from MMI to PTU in relation to pregnancy start. Notably, all studies were register-based and relied on an indirect measure of exposure which challenges the exact assessment of the type and timing of exposure around the pregnancy start. Thus, not only information on double exposure, but also the type of exposure in the period before and after pregnancy start is important to evaluate [32]. A more detailed evaluation of double exposed cases has indicated that the duration of MMI exposure in early pregnancy is critical [8]. This finding may favor a shift in therapy prior to pregnancy also given that the critical window of exposure is from pregnancy week 6 to 10 [8]. Thus, it may be difficult to arrange a shift in therapy after pregnancy detection and prior to this critical period.

**Table 3:** Observational studies on the use of both Methimazole and Propylthiouracil in early pregnancy and outcome of birth defects

Author	Year	Country	Data source	Exposed (n)^a^	Outcome in non-exposed (%)	Outcome in exposed (%)	Association^b^
Korelitz et al. [20]	2013	United States	Health insurance database	126	5.9	11.1	Yes
Andersen et al. [15]	2013	Denmark	Nationwide prescription register	159	5.7	10.1	Yes
Lo et al. [21]	2015	United States	Health insurance database	49	4.4	4.1	No
Andersen et al. [18]	2017	Sweden	Nationwide prescription register	66	8.0	6.1	No
Seo et al. [14]	2018	Korea	Health insurance database	1840	5.9	8.0	Yes
Andersen et al. [16]	2019	Denmark	Nationwide prescription register	255	6.7	7.8	No

^a^Number of children exposed to both Methimazole and Propylthiouracil in the early pregnancy, see text for details

^b^Indicates whether an association between exposure to both Methimazole and Propylthiouracil and birth defects was observed, see text for details

In addition to the choice of treatment in early pregnancy, a pertinent question is on the choice of treatment after the embryonic period of early pregnancy. A general concern about the use of PTU in non-pregnant individuals has been the risk of fulminant liver failure. Such concern has favored the use of MMI, particularly in children [33]. Only few observational studies addressed the risk of liver failure associated with the use of ATDs in pregnant women specifically [21, 34, 35]. Overall, this severe side effect was rarely seen in pregnant women, and in the Danish study [35], birth defects was the predominant side effect to the use of ATD in pregnancy as compared to both liver failure and agranulocytosis. At present, the preferred choice of treatment after the first trimester of pregnancy remains uncertain [2–4]. The immunological changes during a pregnancy characterized by immunosuppression may attenuate the hyperthyroidism of GD and the indication for treatment and the dose of treatment should be carefully monitored with swift adjustments to meet the change in need. Along with considerations on the type and severity of side effects, a shift in therapy may introduce a risk of less controlled hyperthyroidism. Clearly, more evidence is needed, and studies with detailed assessment of the type and timing of exposure are warranted to inform the clinical guidance on the when and how to treat maternal hyperthyroidism throughout the pregnancy [32].

Considering alternative treatments, a focus on the feasibility of using potassium iodide has emerged from studies in Japan [36, 37]. In a cohort of 283 pregnant women with GD, treatment had been switched from MMI to potassium iodide in early pregnancy due to intolerance to PTU [36]. These cases were retrospectively reviewed, and the prevalence of birth defects was evaluated according to treatment. In this study [36], the prevalence of birth defects was lower in the group treated with potassium iodide (*n* = 4 of 260; 1.5%) as compared to the group treated with MMI (*n* = 47 of 1134; 4.1%), *p* < 0.05, including a lower prevalence of the severe malformations previously described as part of the MMI embryopathy. There was no difference between the two groups in terms of gestational age at birth, birth weight or perinatal losses, but the frequency of early pregnancy loss was higher in the MMI group (*n* = 164 of 1333; 12.3%) as compared to the iodine groups (*n* = 15 of 283; 5.3%), *p* < 0.05. The authors speculated whether this finding was related to maternal thyroid function in early pregnancy [36]. A general concern about the use of iodine for the treatment of hyperthyroidism is that this treatment may be less effective than ATDs in the control of hyperthyroidism [6]. In a subsequent study from Japan [37], focus was on thyroid function in pregnant women who shifted from MMI to potassium iodide. In a cohort of 240 pregnant women, nearly half of the patients continued treatment throughout pregnancy and around 10% could not have their hyperthyroidism controlled solely by potassium iodide [37]. From a clinical point, the identification of these patients is important considering the choice of treatment, but no specific predictors of this tendency to escape from the antithyroid effect of iodine could be identified in that study [37]. Further studies on the benefits and risks of treatment with potassium iodide in early pregnancy are needed including studies from populations with different levels of iodine intake.

Another pertinent question is the possibility of other treatments or the development of new treatment. This perspective of alternative treatments on the one hand and the proposal of treatment withdrawal in early pregnancy on the other hand call for further research. Clinical studies are needed and basic scientific work is important to support the clinical data and to develop our understanding of the mechanisms by which ATDs cause teratogenic side effects. Furthermore, different types of research serve to determine the role of maternal thyroid function and the role of the autoimmune mechanisms associated with the hyperthyroidism of GD. MMI and PTU are considered to equally cross the placenta [38], to be equally effective in the control of hyperthyroidism in pregnancy [39] and to hold the same risk of inducing fetal hypothyroidism [40]. However, more evidence is needed to unveil the mechanisms underlying their teratogenic potential. Many possible mechanisms are in play, and one may also speculate on the possible interaction between ATD, maternal and fetal thyroid function, and thyroid autoimmunity and how these various exposures may have direct or indirect adverse effects.

## Conclusion

A link between the use of ATDs for the treatment of hyperthyroidism in early pregnancy and birth defects has been brought forward over the past 50 years, and large observational studies have quantified the risk of birth defects associated with different types of ATDs in more recent years. Current evidence substantiates a risk of birth defects associated with the use of MMI and these malformations may be severe. On the other hand, further studies are needed to settle the teratogenic role of PTU and the role of maternal thyroid function and thyroid autoimmunity. Detailed clinical data on the timing and type of exposure in early pregnancy are needed to inform clinical practice on the choice of treatment and the possibility of treatment withdrawal in selected patients. Furthermore, basic scientific work is important to address the mechanisms underlying the development of birth defects, and to precede the development of alternative or new treatments with less severe side effects.

## Data Availability

Data sharing is not applicable to this article as no datasets were generated or analysed during the current study.

## Abbreviations

ATDs: Antithyroid drugs
CMZ: Carbimazole
CI: Confidence interval
GD: Graves’ disease
ICD: International Classification of Disease
MMI: Methimazole
PTU: Propylthiouracil
US: United States
